# Medical centres for the homeless in Hamburg – consultation reasons and diagnoses compared to primary care patients in the regular health care system

**DOI:** 10.1186/s13690-023-01198-w

**Published:** 2023-10-27

**Authors:** Carolin van der Leeden, Hanna Kaduszkiewicz, Sigrid Boczor, Thomas Kloppe, Benjamin Lohmann, Tina Mallon, Anja Rakebrandt, Martin Scherer

**Affiliations:** 1 Department of General Practice and Primary Care, University Medical Centre Hamburg-Eppendorf, Hamburg, Germany; 2 Institute of General Practice, Medical Faculty of Christian-Albrecht-University, Kiel, Germany

**Keywords:** Homeless, Health care utilization, Primary care, Consultation reasons, Diagnoses

## Abstract

**Background:**

In Germany, homeless people are entitled to health care within the regular health care system. However, due to their specific living conditions they make little use of these services. In 2013, three Medical centres for the homeless (MCH) were opened in Hamburg to provide general health care. This study aims to analyse the consultation reasons and diagnoses prevalent among the homeless in comparison to regular primary care patients. It also examines the means and obstacles of integrating the homeless into Germany’s regular health care system.

**Methods:**

From 2013 to 2014, routine medical data of all patients of the MCH consenting to participate in the study were analysed descriptively, in particular consultation reasons (categorised by ICPC-2), ICD-10 diagnoses and data on health insurance status and the use of the regular health care system. Consultation reasons and diagnoses of homeless patients were compared descriptively with data from regular general practices. Additionally, anonymous data on patient numbers, gender and insurance status was exported from the MCH’s software and analysed descriptively for the years 2013 to 2020.

**Results:**

A total of 840 homeless patients in 2013 and 2014 gave consent to the evaluation of consultation reasons and diagnoses. The most frequent consultation reasons in the MCH in 2013 were skin conditions (24%), musculoskeletal conditions (16%) and psychological disorders (14%), in GP practices these were musculoskeletal conditions (22%), conditions affecting the digestive system (14%) and skin conditions (12%). Essential (primary) hypertension, diabetes mellitus type 2 and back pain are among the top-10-diagnoses in GP practices, as well as in MCH. With regard to the other top-10-diagnoses, there are clear differences between GP practices and MCH: “Psychological behavioural disorder due to alcohol” and diagnoses in connection with trauma, skin infections and acute respiratory infections stand out in MCH. 35% of the homeless patients reported a lack of health insurance as the reason for “not making use of” the regular health care system, while 10% reported they were unable to visit a regular general practitioner due to physical or psychological reasons. In the years 2013–2020 46% to 73% of the 8.380 MCH patients had no health care insurance.

**Conclusion:**

Patients consulting the MCH suffer from medical conditions typical for the homeless, namely skin diseases, wounds, injuries and behavioural disorders due to alcohol abuse, but also from “typical” symptoms in regular GP care as cough or lower back symptoms. Consultation reasons mostly are acute illnesses. Chronic diseases are equally present in regular GP and MCH patients, but pose a great challenge for the homeless among other things due to their irregular contact with the health care system. The lack of health insurance poses the greatest hurdle to the integration of the homeless into the regular health care system.

**Table:** 

Text box 1. Contributions to the literature
• Research has demonstrated the importance of meeting the specific needs of the homeless. This study is the first to analyse routine data on consultation reasons and diagnoses of homeless patients in Medical centres for the homeless (MCH) including the patients’ insurance status.
• The findings of this study address existing gaps in the literature by presenting analyses of consultation reasons (ICPC-2), diagnoses (ICD-10), and socioeconomic information of the homeless presenting in MCH. They provide direct input for policies aiming to improve medical care.
• The results contribute to the expansion of knowledge related to milieu-specific diseases, show differences and similarities of homeless and regular primary care patients and why homeless people are unlikely to use the regular health care system.

## Background

Hamburg is the capital of homelessness in Germany. A total of 262,600 homeless people were estimated as living in Germany in 2022 [1], 19,000 in Hamburg alone [2]. Also, the numbers of unrecorded cases and of people without health care insurance are high. In Germany, the number of uninsured individuals is estimated at 61,000 by the German Federal Statistical Office, but experts refer to about 800,000.

In accordance with German law, every person is entitled to health care within the standard health care system, including the homeless. Uninsured people receive medical treatment in case of severe pain or in a life-threatening situation, only. In any other situation, the insurance status has to be provided within 10 days otherwise patients will be charged for health care services privately. Homeless people have difficulties to be insured for different reasons: they may avoid contact with the insurer, are not able to pay the insurance premium or are simply disorganized. Due to their specific life circumstances, preferences and their characteristic response to medical care and illness, homeless people also tend not to seek recourse to the regular health care system when they become sick [3]. As Trabert et al. (2016) [4] pointed out, homeless people have extraordinary health burdens, face diverse health risks and a high mortality risk [5–10]. They are also three times more likely to suffer from chronic diseases compared to the rest of the population [11]. Severe progression of disease and serious complications arise as a direct consequence of homeless people not using the regular health care system [12].

In response to a 2009 study of homelessness in Hamburg [13] the Hamburg Authority for Labour, Social Affairs and Integration (BASFI) and the “Freie Wohlfahrtspflege” (the federation of independent charities) founded the project “Ways out of Homelessness” involving several working groups. In March 2011, the working group “Health Assistance Provision for the Homeless” developed the first proposals for Medical centres for the homeless (MCH). These proposals were subsequently concretised and the project further developed in collaboration with the health insurance schemes and the Hamburg Association of Statutory Health Insurance Physicians. In 2013, three Medical centres providing basic health care for the homeless were set up in Hamburg. These were located in places regularly frequented by homeless people (e.g., next to overnight hostels for the homeless or close to the main railway station). In these centres general practitioners (GPs) provide health care once or twice a week in sessions lasting two to three hours. These consultation sessions are supported by medical assistants or nurses, with social work support also provided on site.

An evaluation report commissioned by the City of Hamburg in 2018 stated: “An appraisal of social care and health studies in this area shows that there are various factors that cause or are conducive to illness in the cohort of single homeless people.” [2]. Thus far the health issues affecting homeless people in Germany have been inadequately described and research has been confined to the specific point of care provision [14–16]. This prompted Bauer to state: “The striking absence of socio-medical research on homelessness in Germany … [contributes] to the perpetuation and chronic character of homelessness.” [14].

Therefore, this study aims to analyse the consultation reasons and diagnoses prevalent among the homeless in comparison to regular primary care patients. It also examines the means and obstacles of integrating the homeless into Germany’s regular health care system.

## Methods

### Data collection

This study is a full coverage survey. In 2013 and 2014, all patients of the three MCH in Hamburg aged ≥ 18 years were asked to participate in the study by the GP working on site and upon agreeement gave their written consent to analyse their consultation data. For documentation purposes the software x.vianova from Medatixx was used for all patients in all three MCH. The software had been adapted to accommodate certain aspects important to the evaluation (e.g., with respect to the place of residence as “no fixed abode, patient lives on the street”). Diagnoses were directly assigned as ICD-10. Handwritten diagnoses were subsequently coded as ICD-10 diagnoses in the centres’ systems by the GPs or the nurses. In case of consent of the patients to the participation in the study their health insurance status, soziodemographic data (age, gender, coutry of origin), consultation reasons and ICD-10 diagnoses were extracted from the software and analysed descriptively. The freely formulated consultation reasons were categorised according to the International Classification of Primary Care, Version 2 (ICPC-2) in the course of data analysis by the researchers. The ICPC-2 is a WHO-recognised medical classification specifically developed for the needs of primary care. Unlike ICD-10 it does not include diagnoses but classifies the patient’s reasons for encounters (consultations). Since 2003, the ICPC-2 comprises 17 chapters (A-Z) coding various physiological, medical and socio-psychological reasons for the consultation.

Additionally, patients answered a questionnaire on the use of the regular health care system which had been developed based on two focus group discussions with ten staff members from the three MCH. All focus group participants had been working in homelessness services for many years. They belonged to different professions and included doctors, social workers and medical assistants, as well as health care and nursing staff. The most common reasons for not using the regular health care system by the homeless indicated in the focus groups were included in the questionnaire. These were “not insured”, “no trust in doctors in private practice”, “language barrier”, “fear”, “shame”, “does not feel taken seriously by doctors in private practice”, “only trusts doctors in MCH”, “other reasons” (which allowed for patient input). Multiple answers were possible.

In addition to the data described above, anonymous data on patient numbers, gender and insurance status of the homeless patients consulting the MCH in the years 2013–2020 were exported from the MCH’s software and analysed descriptively. It was not possible to analyse consultation reasons, diagnoses and to administer the questionnaire on the use of the regular health care system in the years 2015–2020 due to the effort involved in providing information and obtaining informed consent for data analysis.

### Data analysis

Data were analysed by researchers of the Institute and Polyclinic for General Medicine, University Medical Centre Hamburg-Eppendorf (UKE). To ensure comparability, only data from the first consultation of each patient were used.

In order to compare the ICD-10 diagnoses of the homeless with the patients of regular GP practices, billing data of the Association of Statutory Health Insurance Physicians of North Rhine and Westfalia were used [15]. In order to compare reasons for consultations according to ICPC-2, which is not standard in Germany, the ten most frequent consultation reasons recorded in GP practices and analysed within the CONTENT study by Kühlein et al. (2010) were used [17]. Statistical analyses were performed with IBM SPSS (version 22) for Windows.

The study has been approved by the Ethics Committee of the Hamburg Medical Association (case reference PV4354) and complies with the ethical standards of the Declaration of Helsinki.

## Results

### Numbers, gender and insurance status of homeless patients treated in the MCH 2013–2020

**Fig. 1:**
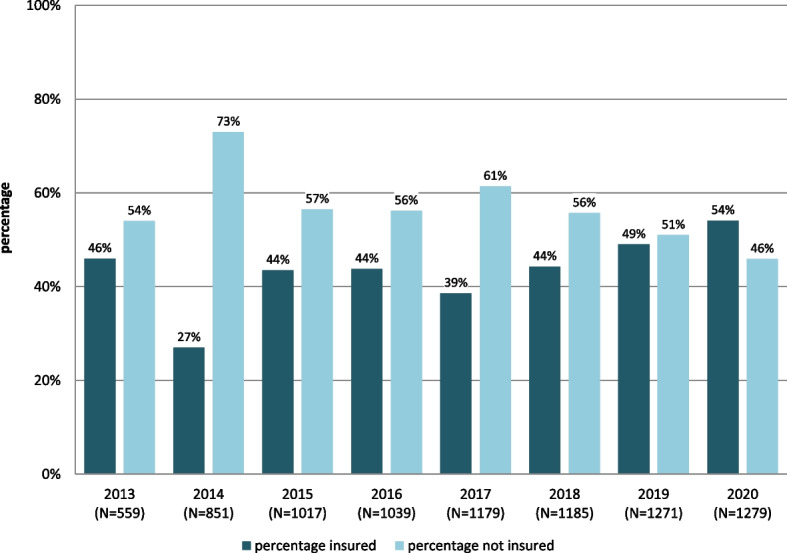
Numbers and insurance status of the patients of the Medical centres for the homeless in Hamburg in 2013–2020. *N* = all patients consulting the Medical centres for the homeless, Hamburg, Germany *N* = 8380

In 2013, 559 and in 2014, 851 patients were treated in the three MCH in Hamburg. The long-term analysis showed a significant increase in the number of patients treated over the years: in 2020 it were 1279 patients. Altogether 8380 patients consulted the MCH in 2013–2020 (see Fig. 1).

In 2013, 13% of patients were female and 87% male; in 2014, the proportion of female patients was 18%, while 82% were male. Considered over the entire period from 2013–2020, 82% of the patients were male and 18% female.

Among the patients in 2013, 54% (*n* = 301) were without health insurance. In 2014, only 27% were insured (*n* = 229). Between 2013 and 2020 the average proportion of uninsured patients was higher (55%) than of insured (45%). The proportion of uninsured patients changed over time (see Fig. 1).

### Analysis of the first two years of the Medical centres for the homeless (2013–2014)

In 2013 and 2014, a total of 1,982 consultations were held with 1,410 patients. In 2013, 559 patients consulted the MCH, of whom 388 (69%) fulfilled the inclusion criteria and consented to data analysis. In 2014, the MCH were consulted by 851 patients, of whom only 452 (53%) consented to data analyses. Consent to participate in the study differed significantly between 2013 and 2014 (*p* < 0.001 in the chi^2^ test). The further evaluations of the years 2013 and 2014 refer to the initial consultations of the total of 840 patients with consent for data evaluation.

Homeless patients attended the MCH between one and 26 times. However, the majority of patients had one (in 2013: 49%, in 2014: 54%) or two consultations (in 2013: 19%, in 2014: 20%). Patient’s age ranged between 18 and 83 years. The mean age was 44 years in 2013, and 43 years in 2014.

In 2013, patients came from a total of 41 countries, 48% of the patients were German. There was a large number of patients from Poland (18%), followed by 5% from Romania. In 2014, 30% stated that they were born in Germany and 68% in another country. Two percent did not give any information on their country of origin. In 2014, patients came from 53 different countries with a large proportion of patients from Poland (22%), Romania (10%) and Bulgaria (6%).

### Reasons for consultations

**Fig. 2:**
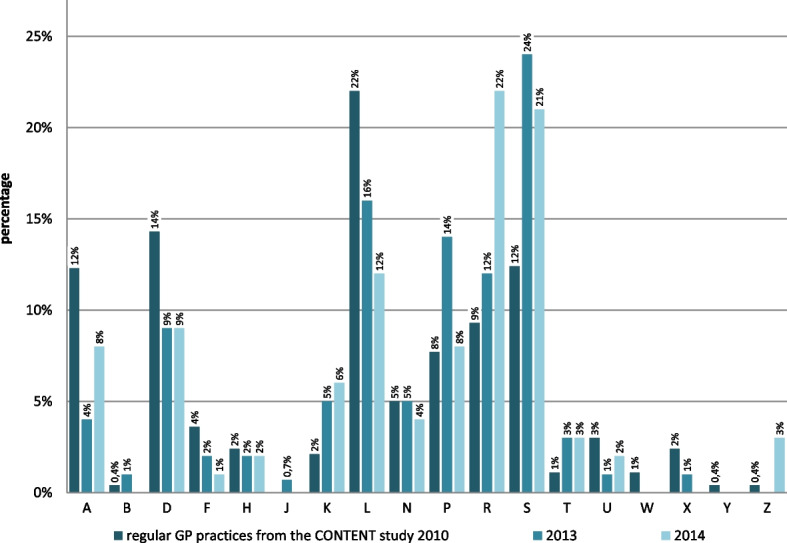
Reasons for consultation in the Medical centres for the homeless in Hamburg in 2013 and 2014 compared to general practice. Data from MCH in Hamburg, Germany, coded according to ICPC-2 for 2013 and 2014. 2013: *N* = 588 consultation reasons (in first consultations of 388 patients); 2014: *N* = 587 consultation reasons (in first consultations of 452 patients). Regular GP practices from the CONTENT study [17]: *N* = 121.677 consultations; ICPC-2 chapters: A General & unspecified, B Blood, haematopoietic organs, lymphatics, spleen, D Digestive tract, F Eye, Ears, K Circulation, L Musculoskeletal, N Neurological, P Psychological, R Respiratory, S Skin, T Endocrine, metabolic & nutritional problems, U Urology, W Pregnancy, birth, family planning, X Female genital system & breasts, Y Male genitalia, Z Social problems

As Fig. 2 shows, the most frequent conditions exhibited by patients in the MCH concern skin (chapter ICPC-2 – S) comprising 24% and 21% of all consultation reasons in 2013 and 2014, musculoskeletal problems (chapter L: 16% and 12%) and psychological problems (chapter P: accounting for 14% and 8% of consultation reasons). By comparison, musculoskeletal conditions are the most frequent reason for consultations in general practice by 22% (Chapter L), followed by 14% of consultation reasons related to the digestive track (Chapter D) and 12% to the skin (Chapter S) [17]. There was a marked increase in respiratory conditions presented in the MCH in 2014 compared to 2013.

When considering the 10 most frequent reasons for consultation cough was equally common in regular GP practices and MCH. Lower back symptoms were comparatively frequent in regular GP practices and MCH in 2013, other frequent consultation reasons differed significantly. Alcohol and drug abuse, trauma and infectious skin conditions, as well as conditions affecting the extremities, especially feet and legs, were found primarily in the MCH cohorts (see Table 1). However, it can be assumed that alcohol and drug abuse were not primary reasons for the consultations of homeless patients but were recorded by the MCH staff. When comparing the MCH consultation reasons between 2013 and 2014, there is an indication of an emphasis on respiratory infections in 2014: cough, throat symptoms/complaints and fever are among the most frequent 5 diagnoses.

**Table 1:** Most frequent reasons for consultation: comparison of patients in Medical centres for the homeless and regular GP practices

Rank	Regular general practice patients	Medical centres for the homeless in 2013	Medical centres for the homeless in 2014
1.	**L03 Lower back symptoms**	**R05 Cough**	**R05 Cough**
2.	**R05 Cough**	P15 Chronic alcohol abuse	R21 Throat symptoms/ complaints
3.	A01: Pain generalised/in several places	P16 Acute alcohol abuse	S02 Itching
4.	D11 Diarrhea	S02 Itching	S19 Skin injury, other
5.	L01: Neck symptoms/discomfort	**L03 Lower back symptoms/discomfort**	A03 Fever
6.	L02: Back symptoms/discomfort, spine/n.s	S19 Skin injury, other	N01 Headache
7.	A23: Risk factors NNB	S18 Laceration/cut injury	K85 Increased blood pressure
8.	P06: Sleep disorder	L17 Foot/toe symptoms/ discomforts	P15 Chronic alcohol abuse
9.	N17: Dizziness/light headedness	L14 Leg symptoms/complaints	L15 Knee symptoms/complaints
10.	F05: Visual disorder, other	P19 Drug abuse	S73 Pediculosis

Reasons for consultation, coded according to ICPC-2; Medical centres for the homeless in Hamburg, Germany: 2013 *N* = 388 patients; 2014 *N* = 452 patients. Regular GP practices from the CONTENT study: most frequent GP consultations of 104,065 patients in the 3-year contact group [17]; Bold notation is used for the comparison of the rankings of the most frequent reasons for consulation

### Diagnoses

In 2013 and 2014 respectively the most frequent ICD-10-diagnoses belonged to the following diagnosis groups: J00-J99 Diseases of the respiratory system (8% and 18%); S00-T98 Injury, poisoning and certain other consequences of external causes (14% and 10%); I00-I99 Diseases of the circulatory system (9% and 14%); F00-F99 Mental and behavioural disorders (11% and 8%); A00-B99 Certain infectious and parasitic diseases (10% and 11%) and M00-M99 Diseases of the musculoskeletal system and connective tissue (10% and 9%). The frequency of all other diagnosis groups was less than 10% in 2013 and 2014.

We compared ICD-10 diagnoses recorded in the MCH with diagnoses from GP practices published by the Central Institute for Statutory Health Insurance Physicians in Germany [18]. Essential (primary) hypertension (I10), diabetes mellitus type 2 (E11) and back pain (M54) are among the most common diagnoses in GP practices, as well as in MCH. With regard to the other diagnoses, there are clear differences between GP practices and MCH. Among the MCH patients the diagnosis “Psychological behavioural disorder due to alcohol” (F10) stands out, ranking first in 2013 and third in 2014. Analogous to the reasons for consultations, the other top-10-diagnoses in the MCH are dominated by diagnoses in connection with trauma and skin infections in 2013, and with acute respiratory infections in 2014. Milieu-specific diagnoses as “early complications of trauma” (T79), “injuries” (T14) as well as “tinea” (B35), “pediculosis” (B85) and “scabies” (B86) are not found among the 50 most frequent diagnoses in regular GP practices. However, the spectrum of diagnoses in MCH is otherwise equally broad and varied as in regular GP practices (see Table 2).

**Table 2:** Most frequent ICD-10 diagnoses in Medical centres for the homeless compared to general practice

Rank	Regular general practice patients	Medical centres for the homeless in 2013	Medical centres for the homeless in 2014
1.	I10 Essential (primary) hypertension	F10 Psych. Behavioural disorder due to alcohol	J06 Acute respiratory infection
2.	E78 Disorders of lipoprotein metabolism and other lipidaemias	**I10 Essential (primary) hypertension**	**I10 Essential (primary) hypertension**
3.	**M54 Back pain**	**E11 Diabetes mellitus, type 2**	F10 Psych. Behavioural disorder due to alcohol
4.	**E11 Diabetes mellitus, type 2**	T79 Early trauma complications	J40 Bronchitis, not designated as acute or chronic
5.	E04 Other non-toxic goiter	**M54 Back pain**	J20 Acute bronchitis
6.	I25 Chronic ischaemic heart disease	B35 Dermatophytosis [tinea]	J44 Other chronic obstructive pulmonary disease
7.	E66 Obesity	T14 Injury n.o.s. of the body region	**M54 Back pain**
8.	**F32 Depressive episode**	B85 Pediculosis/Phthiriasis	**E11 Diabetes mellitus, type 2**
9.	K76 Other diseases of the liver	B86 Scabies	**F32 Depressive episode**
10.	K21 Gastroesophageal reflux disease	K29 Gastritis and duodenitis	B35 Dermatophytosis [tinea]

Coded according to ICD-10; Medical centres for the homeless in Hamburg, Germany: 2013 *N* = 553 diagnoses, 2014 *N* = 451 diagnoses; GP practices: *N* = 600,000 diagnoses, ICD-10 code numbers by specialty group from the Central Institute ADT panel—year 2015 [18]; Bold notation is used for the comparison of the rankings of the most frequent ICD-10 diagnoses

### Reasons for not visiting a GP or the regular health care system on the day of consultation in the MCH

In 2013, 35% of the consulting homeless patients named the absence of health care insurance as reason for using the MCH, while 10% reported they felt physically or mentally unable to attend a regular GP practice. For 6% of the respondents the proximity of the MCH was the reason for chosing treatment there, while 5% each named shame and their financial situation as reasons for not visiting a regular GP.

**Fig. 3:**
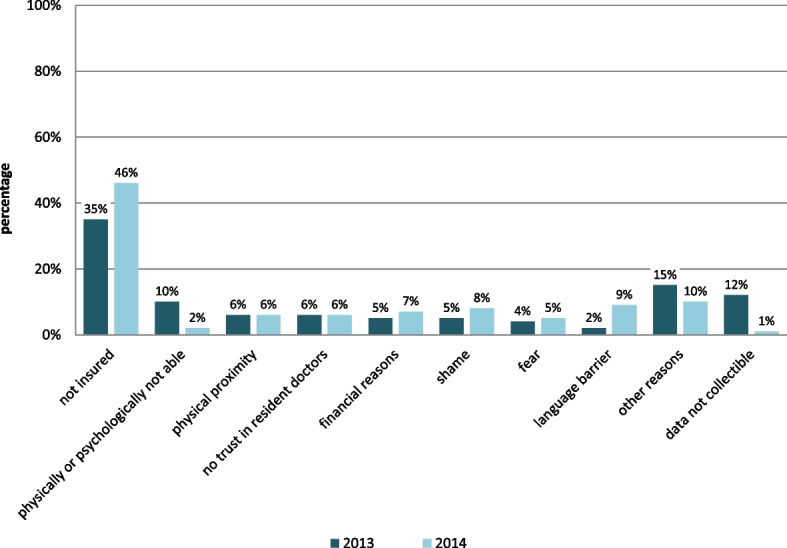
Comparison of reasons for not visiting a GP or the regular health care system in 2013 and 2014. Reasons were given by the patients on a questionnaire, multiple answers were possible, 2013 *N* = 388 with 262 statements, 2014 *N* = 452 with 609 statements

In 2014, 46% of the patients gave the absence of health insurance as a reason for not consulting a GP or the regular health care system (see Fig. 3).

## Discussion

In this study, we evaluated consultation reasons and diagnoses of patients of three Medical centres for the homeless in Hamburg, Germany and compared the results with data from regular GP practices. We also analysed information on health care insurance status of the homeless and reasons for not using the regular health care system.

In the three MCH in Hamburg, which started in 2013, a yearly increasing numer of patients was treated, which underlines the importance of these centres for the medical care of homeless people.

### Age, gender, country of origin and insurance status

In regard to gender, age and country of origin, MCH patients showed similar sociodemographic characteristics compared to other studies of homeless patients [14–16].

In 2013 the average age of the MCH patients was 44 and in 2014 43 years, which corresponds with the average age reported in similar studies of homeless people in Europe [19, 20]. Only in Spain the average age was slightly lower – a little below 39 years [21].

The majority of homeless patients were male. Taken together, 82% of the patients were male and 18% female in 2013–2020. A similar gender distribution was also found in Bertram et al. (2022) [22] and Laere et al. (2009) [23].

MCH patients in 2013 came from a total of 41 countries, in 2014 from 53 countries. Laere et al. (2001) registered homeless people from 30 countries in their homelessness study in Amsterdam [20].

Between 2013 and 2020 the proportion of patients without health care insurance was higher (55%) than of those with insurance (45%). The proportion of uninsured patients changed over time – which might be due to the varying influx of refugees.

### Consultation reasons and diagnoses

A comparison of the reasons for consultations according to ICPC-2 shows that milieu-specific problems such as skin diseases, injuries and alcohol abuse are much more frequently presented by patients in the MCH than in GP practices. As a result, the care of homeless patients is much more time-consuming, e.g., due to behavioural problems, hygiene measures before treatment and the increased need for dressing materials. In addition the analysis of ICD-10 diagnoses reveals, that chronic diseases like essential hypertension and diabetes mellitus type 2 are comparably frequent in MCH and GP practices. Given that the vast majority of patients visited the MCH only once or twice in the respective year and mostly due to acute symptoms, the necessary treatment of their chronic conditions is hardly feasible. White et al. describe reasons for not successful treatment of chronic conditions of the homeless as the patients’ lack of understanding of the disease, lack of self-organisation and of dietary choice, frequent alcohol or drug abuse, and insufficient financial means and/or insurance to cover (not only medication) costs [24].

Other studies also examined the health status of homeless people. For example, Bauer (2012) [14] collected socio-demographic data and ICD-10 diagnoses in a health centre for the homeless in Berlin, Germany. Data can also be found in a study examining the implementation of the concept for “Health Care for Homeless People in North Rhine-Westphalia” [15] and in a historic study by Stößel and Locher (1991) [16].

The comparison of diagnoses with these other studies on the homeless (see Table 3) shows that the frequency distributions are similar. The fact that diagnoses concerning mental health and behavioural disorders (ICD 10, Chapter V) are not always on the top of the list could be explained by the field of specialisation of the health practitioners. For example, a specialist in organic diseases, although noticing mental and behavioural disorders, might code “only” the treatment of acute (organic) diseases for which he or she feels responsible. A study by Jahn and Brönner (2014) in Munich showed that psychiatric disorders requiring treatment had a point prevalence of about 75% among homeless people [25]. This is much higher than in our study in which 16% of patients in 2013 and 8% in 2014 got a diagnosis related to mental and behavioural disorders (these were 11% and 8% of the diagnoses in the respective years). This may point at a tendency among GPs working in the MCH towards assuming alcohol and drug abuse as “normal” for this patient group and therefore not coding it when they came because of another problem. It can also be assumed that a minority of patients affected by alcohol and drug abuse visited the MCH primarily for this reason.

**Table 3:** Comparison of the distribution of ICD-10 diagnoses in the MCH in Hamburg and in other surveys on the diagnoses of homeless patients in Germany

**Rank**	**MCH Hamburg, 2013**	**MCH Hamburg, 2014**	**Bauer** **(2012) **[14]	**Kunstmann (2009) **[15]	**Stößel and Locher** **(1991) **[16]
1.	Injuries, poisoning and certain other consequences of external causes (14%)	Diseases of the respiratory system (18%)	Certain infectious and parasitic diseases (15%)	Mental and behavioural disorders caused by psychotropic substances (13%)	Injuries, poisoning & other consequences of external causes (44%)
2.	Mental and behavioural disorders (11%)	Diseases of the circulatory system (14%)	Injuries, poisoning and other consequences of external causes (15%)	Diseases of the skin and subcutaneous tissue (11%)	Diseases of the musculoskeletal system (40%)
3.	Certain infectious and parasitic diseases (10%)	Certain infectious and parasitic diseases (11%)	Diseases of the respiratory system (14%)	Diseases of the cardiovascular system (7%)	Diseases of the digestive system (32%)

*MCH 2013* 553 diagnoses, *MCH 2014* 451 diagnoses, *Bauer N* = 543 diagnoses, *Kunstmann N* = 31,363 diagnoses, Stößel and Locher, *N* = 342 patients

Some studies of homeless patients showed more diagnoses in the infectious disease classification, while others reported a higher prevalence of dermatological diagnoses [14–16]. This may be due to a classification problem. For example, an infectious skin disease can be roughly classified as both an “infectious” and a “dermatological disease”.

### Comparison with international studies

The diagnoses found in the MCH in Hamburg, Germany, resemble the diagnoses of homeless patients found in international studies [20, 21, 26] (see Table 4).

**Table 4:** International comparison of the three most frequent diagnoses in homeless patients

**Rank**	**MCH Hamburg, 2013**	**MCH Hamburg, 2014**	**Van Laere, et al. Netherlands **[20]	**Alfranca et al. Spain **[21]	**Pribish et al. Florida, USA **[26]
1.	**Psych. behavioural disorders due to alcohol**	**Acute respiratory infection**	Diseases of the skin and subcutaneous tissue	**Essential (primary) hypertension**	**Psychiatric diagnoses (including substance abuse)**
2.	**Essential (primary) hypertension**	**Essential (primary) hypertension**	**Acute respiratory infection**	**Diabetes mellitus, type 2**	**Hypertension**^**a**^
3.	**Diabetes mellitus, type 2**	**Psych. behavioural disorders due to alcohol**	**Psych. behavioural disorders due to alcohol**	Other chronic obstructive pulmonary disease	**Respiratory disease**^**a**^

The three most frequent diagnoses in Medical centres for the homeless in Hamburg, Germany (2013 *N* = 553 diagnoses in 388 patients and 2014 *N* = 451 diagnoses in 452 patients) in comparison with Van Laere et al., 2017 *Netherlands N* = 364 homeless patients, Alfranca et al., 2021 Spain *N* = 492 homeless patients, and Pribish et al., 2019, Florida USA *N* = 183 homeless patients, ^a^107 homeless patients; Bold notation is used for the comparison of the rankings of the most frequent diagnoses

### Lack of health insurance and reasons for not using the regular health care system

Our results show that the lack of health insurance is a major challenge in the integration of homeless patients into the regular health care system. However, other reasons such as shame, fear and lack of trust in doctors of the regular health care system are also present. This reflects the social stigma associated with homelessness [27] and, at the same time, inadequate standards of care provided by doctors treating the homeless. Competing everyday needs [28], e.g., having to find a place to stay, food, clothing, and sanitary facilities [29], which involve time and energy, limited access to transport [30] and limited places to store personal belongings [31] also prevent homeless people from seeking care within the regular health care system. Magwood et al. concluded in their study: “Practitioners [author's note: GPs] were reluctant to care for persons with the lived experience of homelessness, suggesting that the associated social stigma serves as a barrier to health care for this cohort. Participants called for improved ‘training of practitioners to increase knowledge of patient needs and preferences” [32].

The use of the regular health care system and associated barriers are difficult to compare internationally due to the different health care systems in place. In the USA, most health care facilities are privately operated [33]. Barriers to health care use in the US include lack of insurance, high costs of medication and competing priorities such as finding housing or work [26, 29, 33].

The literature from the United Kingdom (UK) shows a slightly different picture. The UK has a state-funded health care system that is free if you are registered with a GP [34]. The complexity of the registration process and the reluctance of GPs to add patients with special needs to their list seem to be the main barriers in the UK [26, 35–37].

In Ghent, Belgium, there is a unique health care system for homeless people. Health centres provide interdisciplinary, comprehensive primary health care for the entire population. Social services bring homeless patients to these centres in a targeted and proactive way. Billing is formally regulated and is not a hurdle for the patients. Uninsured people are treated without any bureaucracy [19].

In the US and UK, there is a lower use of outpatient care in the homeless population and a significantly higher use of hospital and emergency care [29, 38, 39]. In Ghent, the probability of homeless people contacting GPs rather than emergency care is significantly higher [19].

### Strengths and weaknesses of the present study

This study is the first in Germany to analyse routine medical data of homeless patients. The strengths of the study lie in the full coverage survey in the years 2013 and 2014, where valuable data on consultation reasons and diagnoses could be obtained. These show the milieu-specific health problems of the homeless and hence offer a starting point for symptom-related training of professionals. Additonally, the analysis of reasons for not using the regular health care system indicates that the patients’ lack of insurance is a major problem [8].

However, we cannot exclude selection bias as 69% of MCH patients in 2013 and 53% in 2014 gave consent to the evaluation of their data. As we could only report data from one German city, the external validity of the study is limited, too. In addition, we have to assume, that some of the homeless did not seek medical help despite needing it or just focused on urgent somatic complaints. This study therefore does not fully reflect the medical treatment needs of the homeless.

### Implications for the health care system

This study suggests that the homeless require a system of care tailored to meet their specific needs. The long-term goal behind the Medical centres for the homeless in Hamburg was to integrate them into the regular health care system. However, in view of the underlying problems facing the homeless, their particular needs and the lack of health insurance cover, this seems possible only in individual cases. This was also shown by Jego et al. (2018) in their systematic review who concluded: “Homeless need tailored primary care organizations and multidisciplinary team-based models which include primary care physicians, clinic nurses and social worker support. The multidisciplinary team should be trained in special needs of the homeless, their social rights and the supporting system to pass on the knowledge to the patients.” [40].

Due to the milieu-specific characteristics affecting the care and treatment of homeless people, guidelines should be developed to support doctors in their diagnostic and therapeutic approach. Magwood et al. (2020) [32] developed criteria for a “Clinical Practice Guideline for Homeless Health” which addresses the upstream social and health needs, as well as the downstream health consequences of inadequate housing. Specifically, interventions indicated include the provision of supportive housing, income support and case management. Other indicated measures include the provision of supervised consumption rooms and opioid agonist therapy.

“Systematically developed decision-making aids on the appropriate medical approach (…) from which recommendations for action emerge and which would ideally include the so-called risk/benefit and feasibility considerations” [41] are especially important for homeless people as it is precisely the risk/benefit and feasibility considerations that differ massively in comparison with regular GP patients.

Finally, people without health insurance have the right to obtain medical care. This awareness needs to be built up and strengthened among the homeless and all health care providers.

### Implications for further research

With regard to the regular implementation of Medical centres for the homeless, it is important to examine the consequences for health budgets. In recent decades, an increasing number of health programmes for homeless people have emerged in the Western world with very different approaches. There are, to name just a few examples, concrete medical services for homeless people, support in accessing regular health care or ‘Housing First’ concepts, which also intend to improve the state of health and access to regular medical care for the homeless by stabilising their everyday lives [35, 42–45]. Research results from Australia, Canada and the USA suggest that these programmes are more cost-effective than regular care, reduce hospitalisation and provide important social-medical support [42–48]. Unfortunately, these programmes are not described in detail, so directly transferring them to Germany seems problematic [20, 41]. A description and ideally a comparative evaluation of the different care concepts is necessary.

In common with many other studies of this kind, this study is a cross-sectional observation study. Information on the development of the health status of homeless people over time, the impact of structured low-threshold health care as well as opportunities and obstacles to improve health outcomes are still to be investigated. Future studies should therefore particularly examine long-term developments in order to identify the steps needed to significantly improve the health of homeless people.

## Conclusion

Patients consulting the MCH suffer from medical conditions typical for the homeless, namely skin diseases, wounds, injuries and behavioural disorders due to alcohol abuse, but also from “typical” symptoms often seen in regular GP care as cough or lower back symptoms. Consultation reasons mostly are acute illnesses. Chronic diseases are equally present in regular GP and MCH patients, but pose a great challenge for the homeless among other things due to their irregular contact with the health care system. The lack of health insurance poses the greatest hurdle to the integration of the homeless into the regular health care system. Other reasons include amongst others their milieu-specific diseases with special treatment needs, shame, anxiety, language barriers and financial restrictions. Medical care provision for the homeless can only be stabilised in the long term through specialised programmes that take the particular needs of the homeless into account.

## Data Availability

The datasets analysed during the current study are not publicly available due to ethical restrictions involving patient data but are available from the corresponding author on reasonable request.

## Abbreviations

GP: General practitioner
GPS: General practice surgery
BASFI: Hamburg Authority for Labour, Social Affairs and Integration
MCH: Medical centres for the homeless
UKE: University Medical Centre Hamburg-Eppendorf
ICPC-2: International Classification of Primary Care
ICD-10: International Statistical Classification of Diseases and Related Health Problems
